# Specific Stimuli Induce Specific Adaptations: Sensorimotor Training vs. Reactive Balance Training

**DOI:** 10.1371/journal.pone.0167557

**Published:** 2016-12-02

**Authors:** Kathrin Freyler, Anne Krause, Albert Gollhofer, Ramona Ritzmann

**Affiliations:** Department of Sport and Sport Science, Albert-Ludwigs-University of Freiburg, Freiburg, Germany; Northwestern University Feinberg School of Medicine, UNITED STATES

## Abstract

Typically, balance training has been used as an intervention paradigm either as static or as reactive balance training. Possible differences in functional outcomes between the two modalities have not been profoundly studied. The objective of the study was to investigate the specificity of neuromuscular adaptations in response to two balance intervention modalities within test and intervention paradigms containing characteristics of both profiles: classical sensorimotor training (SMT) referring to a static ledger pivoting around the ankle joint vs. reactive balance training (RBT) using externally applied perturbations to deteriorate body equilibrium. Thirty-eight subjects were assigned to either SMT or RBT. Before and after four weeks of intervention training, postural sway and electromyographic activities of shank and thigh muscles were recorded and co-contraction indices (CCI) were calculated. We argue that specificity of training interventions could be transferred into corresponding test settings containing properties of SMT and RBT, respectively. The results revealed that i) postural sway was reduced in both intervention groups in all test paradigms; magnitude of changes and effect sizes differed dependent on the paradigm: when training and paradigm coincided most, effects were augmented (P<0.05). ii) These specificities were accompanied by segmental modulations in the amount of CCI, with a greater reduction within the CCI of thigh muscles after RBT compared to the shank muscles after SMT (P<0.05). The results clearly indicate the relationship between test and intervention specificity in balance performance. Hence, specific training modalities of postural control cause multi-segmental and context-specific adaptations, depending upon the characteristics of the trained postural strategy. In relation to fall prevention, perturbation training could serve as an extension to SMT to include the proximal segment, and thus the control of structures near to the body’s centre of mass, into training.

## Introduction

Balance training is widely applied in different settings of rehabilitative medicine [1], geriatrics [2,3] and as a training method for fall and injury prevention [4–6]. The generic term “balance training” comprises all exercises on instable or moveable surfaces in manifold training settings. Within this, typically two types of balance modality can be subdivided: static and reactive balancing. These two balance types can be classified according to their biomechanical constraints and differ in how instability is induced, thus they result in different motor strategies of compensating for dislocated masses: Importantly, SMT (executed on wobbling or tilt boards, soft mats, cushions, etc.; [7,8]) can mainly be characterized by a single inverted pendulum model [9–13]. Thereby, the relative mass which needs to be adjusted in regard to the gravitational vector is proportionally large, inducing large torques in the ankle joint. In reactive balance tasks, upright stance is perturbed via translations of support surface. According to perturbed stance, investigations support the concept of a double inverted or ‘multi-link’ pendulum [13–17], whereby during re-adjustment of the body the relative mass is distributed over several joints from the ankle up to the knee and hip joints.

Based on the biomechanical differences of the training modalities, we expect that SMT must not necessarily evoke similar functional and neuromuscular adaptations compared to RBT. Although no intervention studies exist comparing both balance modalities in one common experimental setting, there is evidence in literature that these two training stimuli may cause task-specific adaptations [18,19]. The essential differences in biomechanical characteristics and stabilizing strategies between SMT and RBT are described as follows.

1. From a biomechanical point of view, interventions differ in regard to the “fixed point”, the pivot, and the “mobile point”, the moving components that are accelerated during the postural task: Comparing SMT to RBT, stabilizing torques are shifted from distal to proximal. The fixed point is exchanged from the ankle joint (SMT) to the centre of mass (COM) itself (RBT), and the “mobile point” in turn is exchanged from the whole body COM (SMT) to the feet (RBT) [9,10,13,15,20,21].
2. COM stabilization requires different strategies. Studies investigating static stance refer to balance control focusing on the stabilization of the ankle joint with the target to stabilize the gravitational vector of the COM within small trajectories vertically above the feet [9,20–22]. In contrast, in response to perturbation, the COM is horizontally shifted, inducing larger horizontal head and trunk displacements [23–25]. Hence, RBT requires a multi-joint strategy involving a torso replacement to relocate the vector of the COM within broad critical trajectories adequately above the feet [10,16,26–28].
3. The origin of the postural disturbance differs. In SMT, merely self-initiated disruptions of stability cause the neuromuscular system to generate corrective torques to move the vector of the COP in phase with the vector of the COM within the self-initiated frequency [9,29]. In RBT, postural disturbances are applied externally and equilibrium is disrupted by unexpected stimuli via application of unpredictable, random platform displacements [26,30–33].

Distinctions regarding the different training stimuli described in 1–3 are inevitably associated with peculiarities in neuromuscular control and the distribution of involved body segments. Hence, distinction can be drawn in terms of the specificity of the motor control strategy: Supported by literature, the main contributors to control the COM above the base of support in SMT are distal muscles encompassing the ankle joint [6,9,16,22,34]. Conversely, RBT requires an active re-positioning or “tracing” of the COM according to the feet translation, mainly executed by an intersegmental neuromuscular coordination pattern including thigh and hip muscles [28,35,36].

Based on the above-mentioned aspects, we expect that long-term exposure to SMT compared to RBT leads to highly task-specific adaptations. The rationale of this study was to verify the specificity of adaptations in response to these two balance training modalities based on a test-intervention paradigm. Figuring out such differences may be of significant relevance for the practical application of SMT and RBT in rehabilitation or prevention settings with an impact on balance training recommendations. For a conclusive statement, it was tested whether the specificity of training interventions could be depicted in various test paradigms by quantifying balance performance regarding neuromuscular adaptation, segmental involvement and changes in sway path. For this purpose, the postural requirement profile of each training intervention (SMT or RBT) corresponded with one particular test paradigm. To strengthen the study design, in a third test paradigm (transfer task) it should be tested whether specificity of adaptations is limited to the corresponding test paradigm itself [37–40], or if they can be transferred to another, not trained paradigm. We hypothesize that both regimens improve balance performance in all paradigms. However, we expect adaptations to be specific with particularly high effects in the respective paradigm in which postural demands coincide with the trained exercise. Equal effects for both groups are estimated when transfer skills are required in the transfer paradigm.

## Materials and Methods

### Subjects

Thirty-nine healthy subjects (23 females, 15 males, 24±3years) participated in the study. The sample size was estimated by means of a power analysis (f = 0.25; alpha = 0.05; power = 0.90) prior to the experiment. Participants had no acute injuries or neurological irregularities and were not allowed to perform any balance training in addition to the study’s schedule during the period and for six months before the start of the experiment. All subjects gave written informed consent to the experimental procedure, which was approved by the ethics committee of the University of Freiburg in accordance with the latest revision of the Declaration of Helsinki. To provide comparable baseline values in both groups, participants were randomly divided by “matched-pairs” into either a reactive balance training group (RBT-group, 11 females, 8 males, 24±3years, height 173±7cm, weight 67±12kg), or a control group performing conventional sensorimotor training (SMT-group, 13 females, 7 males, 24±3years, height 172±9cm, weight 67±10kg). Matched pairs were built based on the postural sway measures (described in detail in the outcome measures). A pair of subjects with almost equivalent performance levels prior to training start was equally distributed to the SMT and RBT group.

### Training intervention

**Fig 1:**
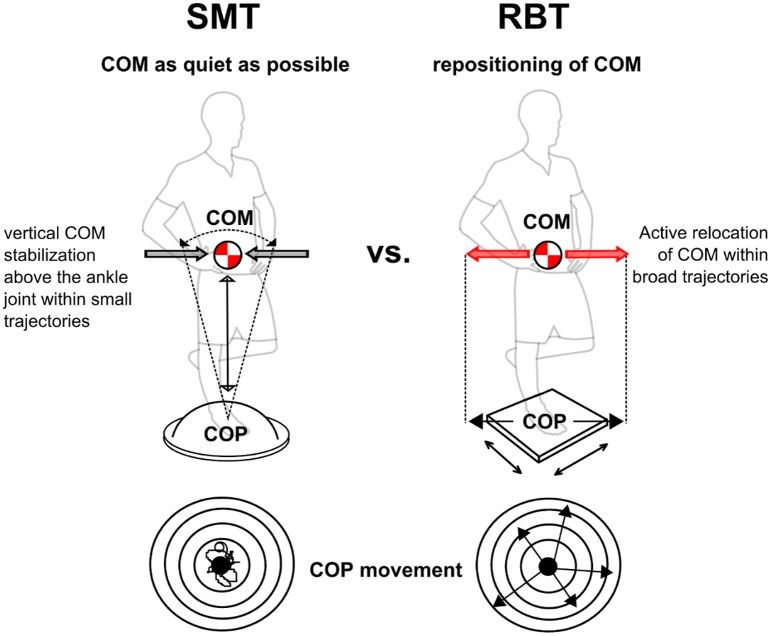
Biomechanical characteristics of the two selected balance trainings: the sensorimotor training (SMT, left) and reactive balance training (RBT, right) intervention. During SMT, the center of mass (COM) needs to be kept as still as possible and stability is reached when the COM stays solidly with the body as an inverted pendulum rotating around the ankle joint. During RBT, after each perturbation of the support surface, the COM needs to be actively relocated through the proximal segment to keep a stable equilibrium.

Both biomechanical paradigms of the training interventions are described in Fig 1. Both training interventions lasted four weeks and comprised three sessions per week with a duration of twenty minutes each. One session comprised four sets separated by one minute rest interval: each set contained four repetitions, respectively. Each repetition lasted one minute and was divided into 40 seconds training/20 seconds intermittent rest interval [8]. RBT was performed on an electromagnetically driven swinging platform (40x40cm, Perturmed^®^; for further information about the device, see [41]), generating random surface translations in the horizontal plane (Fig 1). To eliminate anticipation of perturbations during RBT, perturbations were randomly varied in (i) direction (medio-lateral [m-l], anterior-posterior [a-p] and diagonals), (ii) order of applied direction (not randomised vs. randomised), (iii) amplitude (2, 4, 6, 8cm), (iv) order of applied amplitude (not randomised vs. randomised), (v) duration of platform remaining in perturbed position (100, 275, 1500ms), (vi) pause length between perturbations (1, 2, 3, 4s), (vii) order of the pause length (not randomised vs. randomised), embracing a training matrix with 16 levels in total. The subjects had the instruction to stabilize equilibrium as fast as possible after perturbation. After the initial platform displacement, the platform described a damped sinus curve until subjects reached a stable equilibrium (within ~1.5s), before the next perturbation was triggered. SMT [7] was performed with conventional balance devices including unstable surfaces such as foam balance pads (Airex^®^) or balance cushions (Togu^®^ Dynair air cushions ø33cm; Aero-Step cushions 51x37x8cm/46x32x8cm; Jumper^®^ 52x24cm, BOSU^®^ 67x65x22cm), so that the complexity of balance performance ranged from easy to more challenging postural demands. Subjects had the instruction to stand as still as possible. For both groups, the level of performance difficulty was increased gradually and individually during training by changing to a more challenging perturbation programme during RBT, such as increase of translation displacement, increase of additional directions, increase of the length of perturbation stimulus (holding the platform up to 1.5s at the perturbed end position) and reduction of durations between signals, or by changing to a more challenging support surface during SMT and subsequently by closing the eyes to exclude visual cues. All training sessions were surveyed, supervised and documented. Training was executed with the left leg. Training load of both interventions was matched regarding duration, frequency and rest interval of training periods and sessions.

### Test paradigms

**Fig 2:**
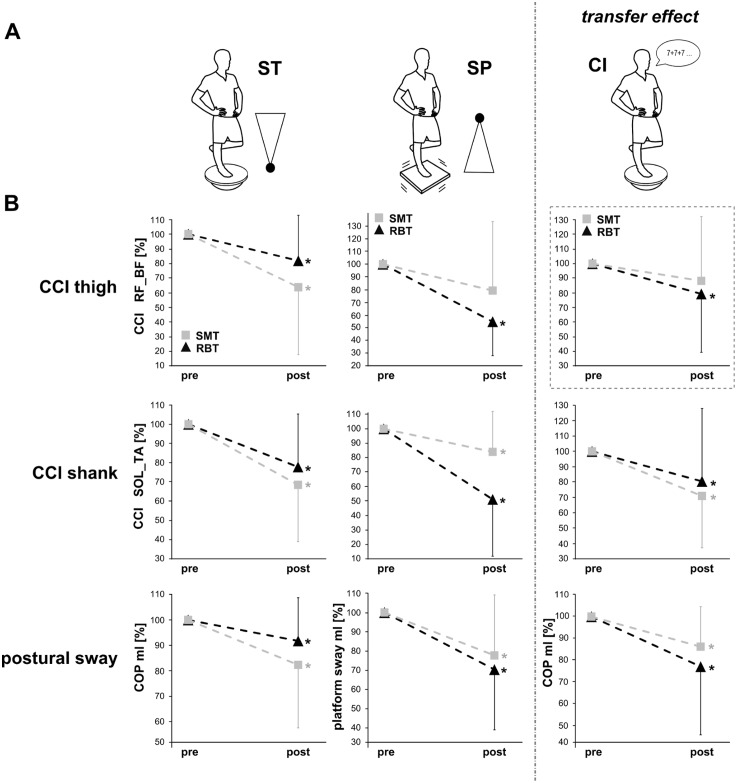
Changes in neuromuscular activation and postural sway in response to both interventions. (B) Changes in thigh (upper row) and shank (middle row) CCIs as well as COP_ml_/postural sway (bottom row) in response to the training interventions SMT (grey square) and RBT (black triangle) in (A) the three different test paradigms on the spinning top (ST, left column), on the swinging platform (SP, middle column) and during the transfer condition with cognitive interference (CI, right column). Adaptations in all parameters were greater in the SMT-group than in the RBT-group for ST, whereas during SP, adaptations were greater in the RBT-group than in the SMT-group. For CI, a greater reduction in COP_ml_ displacement was observed in the RBT-group: it is assumed that the higher decrease in thigh CCI (dashed box) led to the better functional balance performance.

The test-intervention specificity of adaptations in response to four weeks of SMT vs. RBT was evaluated on the basis of a longitudinal repeated-measures matched-subject design. We examined whether specificity of training interventions could be transferred into corresponding test settings. For this purpose, the postural requirement profile of each training intervention (SMT or RBT) corresponded with one particular test paradigm (protocol 1 and 2), a third protocol served as a transfer test (shown in Fig 2A). To strengthen the study design, we chose test settings that none of the groups had trained in.

For all protocols, participants were balancing barefoot on the left leg and instructed to place their hands on their hips, direct their head and eyes forward and to stand as still as possible. Three trials of 30 seconds were performed for each protocol, separated by 30 seconds of rest. The order of the three protocols was conducted counter-balanced across participants but was identical for each subject for pre/post measurements; between each protocol, subjects rested for three minutes. Ankle, knee and hip joints were supervised visually to control the body position. To exclude habituation effects, prior to measurements subjects practised for a period of 10 minutes to adapt to the different surfaces. For the assessment of balance performance, functional and neuromuscular adaptations were quantified by postural sway as well as co-contraction index of antagonistic shank and thigh muscle pairs.

#### Protocol 1—spinning top (ST)

Subjects had to balance on the left leg on a spinning top (ST; Fig 2A). This test protocol focused on static stability to assess adaptations in a test condition corresponding to the postural strategy of SMT (fixed point at the ankle joint) [7,42–44]. By standing as still as possible on the unstable support surface, subjects had to produce and compensate self-initiated postural control manoeuvres. We paid particular attention that this device was not exercised during the four week SMT intervention.

#### Protocol 2—swinging platform (SP)

Subjects had to balance on the left leg on a free-swinging platform (SP; Fig 2A). This test protocol focused on the postural requirement profile similar to RBT (fixed point in the proximal body part) [16,17], challenging the subjects to maintain equilibrium on a freely oscillating support surface. This device (Posturomed^®^, Haider Bioswing, Germany) is frequently used to assess balance performance with high test-retest reproducibility and consists of a platform attached to a solid frame via two steel ropes on each corner [45–47]. Subjects were instructed to stand as still as possible were not exposed to perturbations.

#### Protocol 3—Transfer task with cognitive interference (CI)

This task was applied to establish whether there is any transfer effect of specificities regarding balance performance using cognitive interference (CI; [39,40,48]). Cognitive interference during postural demanding tasks—frequently occurring in everyday life—is known to comprise performance capacity and is related to an increased fall risk [5,49–51]. Subjects balanced on the left leg on a spinning top while performing a cognitive dual task (Fig 2A). CI was applied by means of simultaneous mental calculation (maths summation task; [39,49,52]). Subjects had to count in steps of seven starting from a number randomly chosen by the operator. Instructions were given to count as quickly and accurately as possible.

### Outcome measures

#### Postural sway

Postural sway during ST and CI was quantified by means of the centre of pressure (COP) displacement on a force plate (AMTI, Watertown, USA). 3D ground reaction forces were sampled at 50Hz, and total (COP_total_), anterior-posterior (a-p, COP_ap_) and medial-lateral (m-l, COP_ml_) COP displacement was determined. Sway path of the swinging platform (SP) was assessed by means of a movable goniometer attached to the platform and quantified in a-p and m-l direction. COP and sway path were used to allocate subjects by matched pairs either to the SMT or to the RBT group.

#### Electromyographic (EMG) activity

Bipolar Ag/AgCl surface electrodes (Ambu Blue Sensor P, Ballerup, Denmark; diameter 9mm, centre-to-centre distance 25mm) were placed over the soleus (SOL), medial gastrocnemius (GM), tibialis anterior (TA), rectus femoris (RF) and biceps femoris (BF) of the left leg. The longitudinal axes of the electrodes were in line with the presumed direction of the underlying muscle fibres according to SENIAM [53]. The reference electrode was placed on the patella. Interelectrode resistance was kept below 2kΩ by means of shaving, light abrasion and degreasing of the skin with a disinfectant. The EMG signals were transmitted via shielded cables to the amplifier (band-pass filter 10Hz to 1kHz, 1000x amplified) and recorded with 1kHz (A/D-conversion via a National Instruments PCI-6229 DAQ-card, 16bit resolution). Preliminary isometric maximal voluntary contractions (MVC) were performed for all recorded muscles according to Roelants et al. [54] for normalization. Co-contraction indices (CCIs) were calculated for the antagonistic muscles GM_TA, SOL_TA and RF_BF as the CCI is an indicator for balance ability reflecting the coordination of the task specific muscle activation and hence the functional level of intermuscular coordination [55–57].

### Data processing

30 seconds of COP_ap_ and COP_ml_ (for ST and CI) and sway path in a-p and m-l direction (for SP) were assessed. Data were averaged for trials and subjects. Post values were normalized to the corresponding values of the pre-test.

For each of the recorded muscles in the three protocols, EMG signals with a length of 30 seconds were rectified, integrated, averaged over the three trials (iEMG [mVs]) and normalized to the individual MVC. Further, to assess the simultaneous activation of antagonistic muscles encompassing the ankle and knee joint as a measure for the ability to adapt intermuscular coordination during balance performance task-specifically [55–57], CCI was calculated with the rectified EMG normalized to the individual MVC by means of the following equation for each antagonistic muscle pair [58]: CCI_i_ = (EMG_i_ of the muscle which was lower activated / EMG_i_ of the muscle which was higher activated) x (EMG_i_ of the muscle which was lower activated + EMG_i_ of the muscle which was higher activated) for each sample point i [0–30.000], CCI = Σ CCI_i_.

### Statistics

To determine differences in the adaptations between the two groups [RBT-group vs. SMT-group] and the two time points [pre vs. post] a repeated measures analysis of variance (*rmANOVA*) was conducted for each of the three tasks (ST, SP and CI) for the dependent variables CCI (GM_TA, SOL_TA, RF_BF) and COP (COP_ap_, COP_ml_)/sway path (a-p, m-l), respectively. If the assumption of sphericity measured by Mauchly's sphericity test was violated, Greenhouse-Geisser correction was used. The level of significance was defined at P<0.05. Further, to elucidate possible specificities between the respective training intervention in adaptations of the above mentioned parameters, eta squared (η^2^) was calculated as an effect size for the pairwise comparisons of the repeated measure factor *time* (with the interpretation of the size according to Cohen [59]: small: 0.01, medium: 0.06, large: 0.12). To detect training effects for each training group, T-tests were used for pairwise comparisons [pre vs. post] with the level of significance at p<0.05. To correct for multiple testing, we used Bonferroni; each p-value (p_i_) for each test was multiplied by the number of tests (p_i adjusted_ = p_i_ * n, n = number of tests). If p_i adjusted_ was <0.05, we considered the respective test *i* to be of statistical significance. Statistical methods were conducted with the statistical software SPSS 20.0 (SPSS, Inc., Chicago, IL, USA).

## Results

Grand means of the postural sway and the CCIs are displayed in Table 1 for ST, Table 2 for SP and Table 3 for CI; percentage changes in postural sway and in CCIs in response to both training interventions are illustrated in Fig 2B.

**Table 1:** Pre and post values of the co-contraction indices (CCIs) and the centre of pressure displacement (COP) during Protocol 1 on the spinning top (ST) are illustrated for the two groups RBT and SMT.

		ST								
		RBT				SMT				
		pre	post	% change	Eta^2^	pre	post	% change	Eta^2^	*rmANOVA*
**CCI**										
Thigh	RF_BF	**537±516**	**439±521**	-18%*	**η^2^ = 0.13**	**665±342**	**424±189**	-36%*	η^2^ = 0.11	*P = 0*.*001 F(1*,*13) = 16*.*63*
Shank	GM_TA	1551±1104	1337±991	-14%	η^2^ = 0.05	**1685±988**	**1180±989**	-33%*	η^2^ = 0.09	*P = 0*.*03 F(1*,*15) = 6*.*05*
	SOL_TA	**1695±855**	**1317±771**	-22%*	**η^2^ = 0.19**	**1769±1208**	**1209±735**	-32%*	**η^2^ = 0.24**	*P = 0*.*003 F(1*,*17) = 11*.*72*
**COP [cm]**										
	COP_ap_	**102.1±21.4**	**92.8±18.1**	-9%*	**η^2^ = 0.23**	**97.1±20.4**	**82.5±11.7**	-15%*	**η^2^ = 0.20**	*P = 0*.*001 F(1*,*18) = 16*.*21*
	COP_ml_	**118.6±24.4**	**108.9±21.7**	-8%*	η^2^ = 0.08	**114.9±43.9**	**94.6±25.6**	-18%*	**η^2^ = 0.12**	*P = 0*.*001 F(1*,*18) = 16*.*54*

Values represent mean values±standard deviations. A * symbol indicates a significant difference for pre/post comparison (in bold, *P<0*.*05*), *rmANOVA* time effects are listed in italics. For pairwise comparisons, a high effect size (η^2^>0.12) is marked in bold.

**Table 2:** Pre and post values of the co-contraction indices (CCIs) and the platform sway path during Protocol 2 on the swinging platform (SP) are illustrated for the two groups RBT and SMT.

		SP								
		RBT				SMT				
		Pre	Post	% change	Eta^2^	Pre	Post	% change	Eta^2^	*rmANOVA*
**CCI**										
Thigh	RF_BF	**669±924**	**376±354**	-45%*	**η^2^ = 0.38**	581±524	460±333	-21%	η^2^ = 0.001	*P = 0*.*02 F(1*,*10) = 7*.*49*
Shank	GM_TA	**2266±1740**	**1347±874**	-41%*	η^2^ = 0.07	2020±1386	1778±1685	-22%	η^2^ = 0.09	*P = 0*.*03 F(1*,*13) = 6*.*24*
	SOL_TA	**2316±1743**	**1185±540**	-49%*	**η^2^ = 0.23**	**2299±1939**	**1932±2205**	-26%*	**η^2^ = 0.26**	*P = 0*.*01 F(1*,*11) = 9*.*79*
**Sway path [cm]**										
	Sway_ap_	56.4±6.2	50.9±15.2	-10%	η^2^ = 0.03	43.9±10.0	40.7±19.3	-7%	η^2^ = 0.002	*P = 0*.*30 F(1*,*18) = 1*.*11*
	Sway_ml_	**54.7±20.6**	**38.5±8.3**	-30%*	**η^2^ = 0.19**	**53.6±28.7**	**41.7±16.7**	-22%*	η^2^ = 0.08	*P = 0*.*003 F(1*,*18) = 12*.*08*

Values represent mean values±standard deviations. A * symbol indicates a significant difference for pre/post comparison (in bold, *P<0*.*05*), *rmANOVA* time effects are listed in italics. For pairwise comparisons, a high effect size (η^2^>0.12) is marked in bold.

**Table 3:** Pre and post values of the co-contraction indices (CCIs) and the centre of pressure displacement (COP) during Protocol 3 transfer task with cognitive interference (CI) are illustrated for the two groups RBT and SMT.

		CI (transfer task)								
		RBT				SMT				
		pre	post	% change	Eta^2^	pre	post	% change	Eta^2^	*rmANOVA*
**CCI**										
Thigh	RF_BF	**531±419**	**420±353**	-21%*	η^2^ = 0.07	568±441	500±377	-12%	η^2^ = 0.002	*P = 0*.*14 F(1*,*14) = 2*.*49*
Shank	GM_TA	1511±879	1244±507	-18%	η^2^ = 0.003	**1608±990**	**1087±655**	-32%*	η^2^ = 0.07	*P = 0*.*006 F(1*,*17) = 9*.*84*
	SOL_TA	**1703±708**	**1370±543**	-20%*	η^2^ = 0.02	1712±1142	**1215±562**	-29%*	η^2^ = 0.09	*P = 0*.*01 F(1*,*18) = 8*.*00*
**COP [cm]**										
	COP_ap_	101.1±21.3	97.5±19.6	-3%	η^2^ = 0.00	98.5±21.7	89.8±14.1	-9%	η^2^ = 0.02	*P = 0*.*14 F(1*,*17) = 2*.*46*
	COP_ml_	**126.1±32.1**	**97.2±30.6**	-23%*	**η^2^ = 0.17**	**123.8±44.5**	**106.8±25.4**	-14%*	**η^2^ = 0.14**	*P = 0*.*002 F(1*,*17) = 13*.*28*

Values represent mean values±standard deviations. A * symbol indicates a significant difference for pre/post comparison (in bold, *p<0*.*05*), *rmANOVA* time effects are listed in italics. For pairwise comparisons, a high effect size (η^2^>0.12) is marked in bold.

### Postural sway

COP_ap_ displacement was significantly reduced for both groups in the ST protocol; thereby a greater reduction was observed for the SMT-group (-15%) compared to the RBT-group (-9%). COP_ml_ displacement/m-l platform sway was significantly reduced for both groups in all protocols ST, SP and CI: thereby the SMT-group revealed a greater reduction and larger effect sizes in the ST protocol (SMT -18%; RBT -8%) and the RBT-group revealed a greater reduction as well as larger effect sizes in the SP (SMT -22%; RBT -30%) and in the transfer condition CI (SMT -14%; RBT -23%) (Fig 2B). Between group comparison revealed no significant results.

### Co-contraction Index

In both groups in all three protocols (ST, SP, CI), CCIs in the muscles encompassing the ankle joint GM_TA (SMT -33%, -22%, -32%; RBT -14%, -41%, -18%) and SOL_TA (SMT -32%, -26%, -39%; RBT -22%, -49%, -20%) were significantly reduced, with greater effect sizes for SMT, respectively. CCIs in RF_BF were significantly reduced in both groups during ST and SP protocols, with large effect sizes for RBT, whereas the SMT group showed very small effect sizes for thigh CCI reduction in both test protocols. Further, a greater reduction was observed for the SMT-group during ST (SMT -36%; RBT -18%), and for the RBT-group during SP (SMT -21%; RBT -45%) (Fig 2B). Moreover, post hoc analyses revealed that the reduction in thigh CCIs (RF_BF) for both SP and the transfer condition CI was only significant in the RBT-group, whereas the SMT-group showed no significant RF_BF reduction in thigh CCI in these two protocols (see Fig 2B). Between group comparisons revealed no significant results.

## Discussion

The purpose of the study was to ascertain adaption specificities in response to the two types of balance training interventions. Our study revealed three main results. 1) Specific stimuli result in distinct adaptations. Although balance performance improved in both intervention groups in all test paradigms, percentage changes and effect sizes differed dependent on the paradigm: When training and paradigm coincided, effects were augmented. 2) These specificities were accompanied by neuromuscular segmental distinctions with a greater emphasis on the proximal body segment in the RBT-group and on the distal segment in the SMT-group. 3) Larger improvements in balance performance with simultaneous cognitive interference were observed after RBT, indicating augmented transfer effects from one specific training paradigm to another multi-task balance setting.

### Specificity of adaptations and segmental distinction

Although between group comparisons revealed no significant results, effect sizes and percentages clearly represent distinct influences of the two training interventions which point towards specific adaptations regarding the functional performance and the segmental differentiation of neuromuscular adaptations between both groups. Reductions in sway path and COP displacements were enhanced by 11–36% in the respective paradigm in which postural demands corresponded with the trained balance exercise (Fig 2B). These adaptations were emphasized specifically with a greater reduction for the SMT-group during ST and for the RBT-group during SP and CI. According to literature, this result has functional relevance for both groups: reductions in sway paths are associated with a decreased incidence of ankle injuries [60] and a reduced risk of falls [61]. Differences appeared in both directions, m-l and a-p. However, percentage changes in the m-l plane of motion are much more pronounced (Tables 1, 2 and 3). This finding seems of considerable relevance, as Lord et al. [62] have shown that the postural sway in m-l direction displayed a higher correlation with an individual risk of falling.

Moreover, the observed task-specific improvements in functional balance performance are accompanied by a reduced co-contraction of antagonistic muscles. Both intervention groups showed diminished co-contraction of antagonistic muscle pairs after training. As shown by the effect sizes, reductions in contraction indices were pronounced to different extents: regardless of the amount of reduction, for SMT, larger effects of the diminished simultaneously activated antagonistic muscles appear in the distal muscles encompassing the ankle joint, whereas vice versa for RBT effects of diminished co-contractions are more pronounced for the proximal muscles stabilizing the knee and hip joint. This segmental distinction could be observed independent of the test-paradigm (ST or SP). According to previous studies [61,63,55], a high muscle co-contraction reflected by simultaneously activated antagonistic muscle groups points towards a rigid articular stiffening of posture, leading to a general mechanical fixation of that specific limb. Due to this kind of joint stiffening, the ability to react precisely and to rapidly modify movement strategies is restricted, which in turn may lead to greater postural sway [55,64]. Thus, a lower co-contraction is associated with better postural control ability. It is assumed that balance training positively affects the task specific muscle activation which consequently results in an optimized intermuscular coordination [56,57].

### Underlying mechanisms

Regarding the segmental distinction of neuromuscular adaptations, the training specific effects might be based upon the fact that the two training regimens require—at least in part—different motor control strategies to maintain postural stability. Aspects dealing with the biomechanical differences of the training modalities may be substantial for the interpretation of the results: In both modalities, the controlled variable is the COM [20], but vertical stabilization of the COM vector above the ankle joint is achieved differently for SMT compared to RBT. With the focus to stand as still as possible, a typical situation in SMT, the human body mainly acts as a single inverted pendulum. Biomechanically, this type of motor control requires a large torque in the ankle joint, mainly produced by muscles encompassing the distal segment [9,20,44]. In contrast, experiments executed in perturbed upright stance revealed that the proximal segment moves into focus to relocate the COM back to the vertical (Fig 1) [10,16,27]. It is supposed that—in contrast to the distal control during SMT—the control of the trunk is the fundamental strategy to keep equilibrium after perturbation [23,24,65] and stabilizing joint torques are distributed from the ankle towards the knee and hip joints [16,24,35,36].

Consequently, different muscle activation patterns are needed when comparing control of static posture during unperturbed stance and control of static posture while perturbations of the support surface are applied [18,19]: during SMT, when standing as still as possible, balance control is supposed to be predominantly based on proprioceptive sensory input via the Ia afferent pathway evoking a mainly reflex based muscle activation within the shank muscles [7,9,16,34]. As a consequence, and as previously shown in a number of studies, neuromuscular adaptations in response to SMT predominantly occur in distal muscles [6,66], reflected by the reduced co-contraction of antagonistic shank muscles in our results. In contrast, when postural control is challenged by external stimulation (i.e. perturbations of the support surface), it is supposed that proximal muscle activity (i.e. thigh muscles) is associated with the control of trunk orientation to restore equilibrium on the perturbed platform [16,27,28,67,68] and it is assumed that corticospinal pathways via polysynaptic II afferents are involved in the neuromuscular control of postural responses [69–71]. Thereby, thigh and hip muscles are activated to compensate for the higher torques produced by external perturbation by generating appropriate compensatory torques in the knee and hip joints [17,28,35]. As a consequence, as shown in our study for the first time, RBT emphasized neuromuscular adaptations in the proximal leg segment, reflected by a reduced co-contraction of antagonistic thigh muscles.

### Transfer effect

“Multi-tasking” is commonly required to perform a wide spectrum of daily activities [72]. Interestingly, the RBT-group revealed 64 percent greater improvements than the SMT-group in balance performance—especially in m-l direction—when simultaneously interfered with cognitive demands. This finding indicates that transfer effects into dual task settings with CI are greater for RBT than for SMT. CI—identified as a major fall risk factor—is highly relevant for fall prevention [38,73]: restrictions in simultaneous cognitive processing and muscle coordination are interrelated with postural instability and increased incidence of falls [29,74]. Training regimens that counteract these deficits in complex multitask settings are highly applicable for subpopulations suffering from an enhanced risk of fall-induced injuries.

In view of literature [38–40,73], until now, balance performance during CI could only be improved in response to CI training; no effects have been observed for balance training executed without CI. Thus our findings are surprising. We can just speculate about the underlying mechanisms: one major factor could be the different motor control strategies within both training interventions as mentioned above. Whereas during SMT it is supposed that postural control is mainly attributed to subcortical centres and reflex activity [34,66,75–77], there is evidence that during RBT the postural responses could be seen as a particular kind of voluntary movement, which is thought to be under control of the motor cortex and hence under a larger influence of cortical areas suggesting an involvement of transcortical loops [77–82]. In particular, it can be assumed that in addition to spinal pathways during the initial part of the muscular response, within the late reflex component (after 85ms) direct corticospinal pathways contribute to the neuromuscular control of perturbed stance [77,78,80,82]. Based on this, it can be speculated that a balance training which includes the usage of corticospinal pathways—as it holds true for RBT—may result in an improvement of the processing of such circuits. Hence, this improvement may have led to a better capacity for additional cognitive demands—and hence to the better functional improvement within the RBT group. Considering other factors contributing to the larger transfer effect within the RBT-group, we assume that variability and segmental strategy also play a role. First, results from Rhea et al. [83] and Yao et al. [84] indicate that the more variable and complex a stimulus is, the more postural control systems are challenged and the greater is the spectrum of trained manners. As the variability is higher during RBT by applying random surface perturbations varying in direction, amplitude, pause and perturbation length, we assume that the ability to effectively transfer performance gains from the frequently repeated task led to enhanced postural stability in CI. Secondly, neuromuscular control of the proximal segment may also have influenced balance performance positively during CI. An improved muscle synergy accompanied by diminished antagonistic co-contraction in the thigh muscles is associated with an enhanced COM control [15] and thus may have led to an improved balance performance.

Although training settings have been matched for RBT and SMT in terms of duration, frequency and rest interval of training periods and sessions, we cannot exclude possible differences in training load and intensity. Thus, this needs to be mentioned as a limitation of the study.

## Conclusion

Our findings elucidate that adaptations in postural control are multi-segmental and functionally specific, depending on the postural strategy which may predominate to control body COM during training. The specificity of training modalities—all combined under the term “balance training”—shows that it is important to adequately distinguish cause and effect between the respective types of balance interventions. Regarding possible implications, commonly SMT has been described as being beneficial in general to rehabilitate from ankle injuries [6] and to prevent postural deficits [7,85]. Hence, with the focus on the ankle prevention and rehabilitation or chronic ankle instabilities, SMT serves as an adequate postural training intervention [86]. Based on the current findings, RBT could serve as a further expansion to include the proximal segment, and thus the control of structures near to the body’s COM, into training. For instance, RBT could promote the prevention and rehabilitation in athletes and other subpopulations suffering from knee and thigh injuries. Concerning fall prevention, the strategy to precisely control upper body segment oscillations is assumed to contribute to the risk of falling [25,87]. Further, considering fall or injury mechanisms, it can be assumed that RBT addresses anticipatory postural skills more than SMT. Together with the higher ability to transfer performance after RBT, this seems promising: in particular, for risk populations such as older people or patients with neurological deficits such as Parkinson or Multiple Sclerosis, the training of postural corrective responses which occur in typical high fall risk situations might be useful. To conclude, it is suggested to adjust the choice of the adequate training intervention to the specific target group (i.e. for patients, athletes, older people), and to particularly identify the aim of the intervention and which structures want to be addressed, to then involve the specific postural strategy within the training. If no specific aim exists, we recommend using both SMT and RBT in expansion to each other.
